# 6 Fixing the Leaky Pipeline of Burn Nurses: Understanding Students’ Apprehension and Improving Exposure

**DOI:** 10.1093/jbcr/iraf019.006

**Published:** 2025-04-01

**Authors:** Dania Johnson, Claudia Nevarez, Kimberley Magsayo, Leonece Myers, Elaine Terr, Violeta Perez, Justin Gillenwater, Haig Yenikomshian

**Affiliations:** Keck School of Medicine of University of Southern California; Los Angeles General Medical Center Burn Unit; Los Angeles General Medical Center; Los Angeles General Medical Center; Los Angeles General Medical Center; Los Angeles General Medical Center; Los Angeles General Medical Center; Keck School of Medicine of University of Southern California

## Abstract

**Introduction:**

Burn care is a crucial nursing specialty currently facing significant workforce challenges, as many burn nurses are leaving the field and attracting new talent is becoming increasingly difficult. We currently lack a clear understanding of the underlying factors contributing to this trend. This study seeks to illuminate nursing students’ perceptions and attitudes toward pursuing a career in burn nursing.

**Methods:**

As part of a new nursing recruitment program, 20 final semester nursing students were randomly selected to participate in a burn-specific lecture followed by a two-day burns shadowing experience, which included observing burn surgeries, participating in wound care, joining multidisciplinary rounds, and attending outpatient clinic visits. Before initiation of this program, they completed a survey on the following topics: preferred work area post-graduation, previous exposure to burn lectures, quality of those lectures (if applicable), interest in burn nursing and wound care, comfort with burn care, and perceived understanding of burn management.

**Results:**

Of the 20 participants, majority expressed interest working in the ICU (70%), followed by ED (50%), OR (35%), Med/Surg/Tele (20%), and “undecided or other” (20%). Among those considering a career in burn nursing (n=14), the most common motivation was the desire to make a significant impact on patients’ lives (64%), followed by interest in the challenge and complexity of burn care (50%). For those not considering burn nursing (n=8), the most common reasons were a lack of exposure (63%) and concerns about the emotional toll (50%).

Notably 85% of students reported having received no formal lectures on burn care in their education. Consequently, 40% rated their understanding of burn injuries and management as “Average,” 10% as “ Good,” and 50% as “ Poor” or “ Very Poor.” Despite this, students expressed moderate interest in burn care (7.4/10) and a high interest in wound care (8.2/10), though their comfort with burn management was lower (5.5/10).

**Conclusions:**

The results highlight a notable lack of exposure to burn care in nursing education, despite students expressing interest in critical care and a potential inclination toward burn nursing. By recruiting and exposing nursing students to burn care concepts earlier in their training, we can cultivate greater interest and readiness for careers in the field. For next steps, we are currently implementing a comprehensive curriculum focused on burn care, with plans to evaluate its effectiveness in enhancing students’ knowledge, comfort, and interest in pursuing burn nursing.

**Applicability of Research to Practice:**

This study aims to analyze interest and exposure to burn nursing as a career amongst graduating nursing students.

**Funding for the Study:**

N/A

